# Surface Roughness-Induced Changes in Important Physical Features of CoFeSm Thin Films on Glass Substrates during Annealing

**DOI:** 10.3390/ma16216989

**Published:** 2023-10-31

**Authors:** Chi-Lon Fern, Wen-Jen Liu, Yung-Huang Chang, Chia-Chin Chiang, Yuan-Tsung Chen, Pei-Xin Lu, Xuan-Ming Su, Shih-Hung Lin, Ko-Wei Lin

**Affiliations:** 1 Department of Materials Science and Engineering, National Chung Hsing University, Taichung 40227, Taiwan; fengcl@yuntech.edu.tw (C.-L.F.); kwlin@dragon.nchu.edu.tw (K.-W.L.); 2 Department of Materials Science and Engineering, I-Shou University, Kaohsiung 84001, Taiwan; jurgen@isu.edu.tw; 3 Bachelor Program in Industrial Technology, National Yunlin University of Science and Technology, 123 University Road, Section 3, Yunlin, Douliou 64002, Taiwan; changyhu@yuntech.edu.tw; 4 Department of Mechanical Engineering, National Kaohsiung University of Science and Technology, Kaohsiung 80778, Taiwan; ccchiang@nkust.edu.tw; 5 Graduate School of Materials Science, National Yunlin University of Science and Technology, 123 University Road, Section 3, Yunlin, Douliou 64002, Taiwan; lupei1103@gmail.com (P.-X.L.); 54xuanmingsu@gmail.com (X.-M.S.); 6 Department of Electronic Engineering, National Yunlin University of Science and Technology, 123 University Road, Section 3, Yunlin, Douliou 64002, Taiwan; isshokenmei@yuntech.edu.tw

**Keywords:** annealed Co_60_Fe_20_Sm_20_ thin films, low-frequency alternating current magnetic susceptibility (χ_ac_), optimal resonance frequency (f_res_), surface energy, adhesion, nanoindentation, transmittance

## Abstract

Co_60_Fe_20_Sm_20_ thin films were deposited onto glass substrates in a high vacuum setting. The films varied in thickness from 10 to 50 nm and underwent annealing processes at different temperatures: room temperature (RT), 100, 200, and 300 °C. Our analysis encompassed structural, magnetic, electrical, nanomechanical, adhesive, and optical properties in relation to film thickness and annealing temperature. X-ray diffraction (XRD) analysis did not reveal characteristic peaks in Co_60_Fe_20_Sm_20_ thin films due to insufficient growth-driving forces. Electrical measurements indicated reduced resistivity and sheet resistance with increasing film thickness and higher annealing temperatures, owing to hindered current-carrier transport resulting from the amorphous structure. Atomic force microscope (AFM) analysis showed a decrease in surface roughness with increased thickness and annealing temperature. The low-frequency alternating current magnetic susceptibility (χ_ac_) values increased with film thickness and annealing temperature. Nanoindentation analysis demonstrated reduced film hardness and Young’s modulus with thicker films. Contact angle measurements suggested a hydrophilic film. Surface energy increased with greater film thickness, particularly in annealed films, indicating a decrease in contact angle contributing to this increase. Transmittance measurements have revealed intensified absorption and reduced transmittance with thicker films. In summary, the surface roughness of CoFeSm films at different annealing temperatures significantly influenced their magnetic, electrical, adhesive, and optical properties. A smoother surface reduced the pinning effect on the domain walls, enhancing the χ_ac_ value. Additionally, diminished surface roughness led to a lower contact angle and higher surface energy. Additionally, smoother surfaces exhibited higher carrier conductivity, resulting in reduced electrical resistance. The optical transparency decreased due to the smoother surface of Co_60_Fe_20_Sm_20_ films.

## 1. Introduction

Samarium (Sm)-cobalt (Co) permanent magnetic material stands out as an outstanding hard magnet [1]. The optimization of the magnetic performance in Sm-Co alloys often involves the addition of elements such as copper (Cu), iron (Fe), zirconium (Zr), and boron (B) [2,3,4,5]. However, the microstructure and magnetic properties of the alloy are significantly affected by the Co content. [6]. The introduction of Fe into the mix encourages the emergence of soft magnetic phases rich in Fe, characterized by higher magnetic moments. This, in turn, elevates the remanence (Mr) and saturation magnetization (Ms) of SmCo_5−x_Fe_x_ alloys, while simultaneously diminishing coercivity (H_c_) [7,8]. CoFe alloys exhibit outstanding saturation magnetization (Ms) as well as attractive physical properties like magnetostriction (λ) and anisotropy constants. When carefully crafted with suitable compositions and subjected to specific heat treatments, these alloys can serve as top-notch materials for applications in both soft magnetism and magnetostriction. In applications like electromagnetic lenses, electron microscopes, advanced print heads, and linear pulse motors, where reliability is of utmost importance, CoFe serves as a critical component. By manipulating the cobalt-to-iron ratio, the magnetic properties of CoFe can be fine-tuned to meet specific requirements. Furthermore, the substitution of cobalt for iron in appropriate quantities can raise the Curie temperature (T_c_), thereby extending the high-temperature operational range [9,10]. However, it is important to note that during annealing and conventional processing, the material’s tendency to become brittle or to be deformed is notable, and CoFe alloys do not exhibit low coercivity. As the annealing temperature increases, there is a significant reduction in magnetic anisotropy, complicating the task of meeting the high-temperature requirements for these magnetic devices [11,12,13]. Introducing a third element is one viable approach to improving the thermal stability of CoFe alloys [14]. CoFe thin films find widespread applications in magnetic materials and magnetic storage systems [15,16]. However, the addition of Sm elements may further enhance their performance, including improvements in magnetic and structural properties [17,18,19,20]. In past studies, the incorporation of rare earth elements into Fe or CoFe thin films has been investigated to improve their magnetic properties and thermal stability [21,22]. Sm is known for its strong magnetic moments and can enhance the magnetic anisotropy and saturation magnetization [23]. Additionally, Sm incorporation may influence the microstructure and crystallographic orientation of the thin films, thus affecting their overall performance [24]. In the pursuit of enhancing the properties of thin film materials to meet specific application requirements, this study is motivated to investigate the advantages of incorporating Sm in CoFe thin films. Through this research, the aim is to evaluate the impact of Sm-element incorporation into CoFe thin films and explore their potential advantages in terms of magnetic and structural characteristics. However, little research has been focused on Sm addition to CoFe thin films, so exploring the notable properties of CoFeSm films is therefore a valuable pursuit. Specifically, this study focuses on the characterization of Co_60_Fe_20_Sm_20_ thin films as a novel material, with a primary emphasis on evaluating their magnetic properties and exploring their promising applications in magnetism-related domains. Additionally, studies involving Co_60_Fe_20_Sm_20_ thin films are directed toward fine-tuning their attributes to match specific applications, including those in the realm of magnetic sensors [25]. The thickness evaluation is conducted within the range of 10–50 nm, particularly taking into account the typically slender thickness of the free or pinned layer [26]. Substituting a CoFeB seed or buffer layer with CoFeSm film significantly enhances the thermal stability of these materials, making them a more viable choice for real-world magnetic tunneling junction (MTJ) applications. Additionally, under elevated temperatures, magnetic components are markedly affected by environmental conditions, resulting in a notable decrease in magnetic anisotropy at around 350 °C [27]. Given the aforementioned rationales, it is imperative to investigate the distinctive attributes and thermal stability of CoFeSm films across annealing temperatures ranging from 100 to 300 °C. Surface roughness has a significant influence on the incorporation of the magnetic component, whether at room temperature (RT) or during the annealing processes. The physical attributes of ultrathin films are significantly influenced by surface roughness. Academics have delved deeply into roughness concerning magnetic, electrical, and optical properties [28,29]. Specific research reports have observed that the incorporation of rare earth elements can result in modifications of the surface morphology and roughness in TiO_2_ films [30]. These alterations can subsequently exert an influence on the structural, electrical, and optical properties of high-quality CdTe and TiO_2_ films when employing magnetron sputtering deposition techniques [30,31]. The distinctive aspect of this study lies in the integration of Sm into the CoFe alloy and the comprehensive exploration of surface roughness. The principal objective of this research is to establish a direct correlation between surface energy and the magnetic properties exhibited by CoFeSm thin films at varying thicknesses and annealing temperatures.

## 2. Materials and Methods

Sputtering and annealing conditions: sputtering at RT on a glass substrate using a direct current (DC) magnetron sputtering approach, CoFeSm films were created with thicknesses varying from 10 to 50 nm. Investigations were carried out on both as-deposited films and those exposed to annealing treatments at 100, 200, and 300 °C for 1 h in an argon (Ar) environment. The sputtering power was set at 50 W, maintaining a base pressure of 3.7 × 10^−7^ Torr and a working pressure of Ar at 3.2 × 10^−3^ Torr within the vacuum chamber. The Ar flow rate was set at 20 standard cubic centimeters per minute (sccm), and the loader rotated at 20 revolutions per minute (rpm). Post-deposition, the samples underwent annealing for 1 h within a temperature range of 100 to 300 °C, employing a controlled heating rate of 30 °C/min and a cooling rate of 0.5 °C/min. Throughout the annealing process, the vacuum chamber maintained a constant pressure of 2.5 × 10^−3^ Torr.

Compositions: the CoFeSm alloy target was composed of 60 at.% Co, 20 at.% Fe, and 20 at.% Sm. This specific target was a commercially available alloy sourced from pure metals provided by Gredmann Taiwan Ltd. To achieve the desired composition, a powder mixture was prepared, consisting of 99.9% pure Co, Fe, and Sm elements. The target’s composition ratio was validated through certification from the original factory that specializes in composition testing. Elemental composition analysis of the thin films was conducted using an energy dispersive X-ray spectroscopy (EDS, JEOL, Tokyo, Japan) instrument.

Techniques and characterizations: a diverse range of analytical methods was utilized in this study. The crystal structure of the thin films was examined using grazing incidence X-ray diffraction (GIXRD) patterns of Cuk_α1_ (PAN analytical X’pert PRO MRD, Malvern Panalytical Ltd., Cambridge, UK) and low angle diffraction at approximately 2°. Electrical properties were investigated using a four-point probe measurement (Sadhudesign, Hsinchu, Taiwan). Film hardness was determined using nanoindentation with an MTS (mechanical testing and simulation) Nano Indenter XP (KLA, iNano^®^, MTS, Minneapolis, MN, USA) utilizing a Berkovich indenter. Hardness and Young’s modulus were obtained via the continuous stiffness measurement (CSM) technique, ensuring the indenter’s depth of penetration remained less than 10% of the film thickness, and an indentation load of 1 mN was applied. Each sample underwent six indentations, and the results were averaged with a standard deviation for enhanced precision during analysis. Surface roughness and three-dimensional (3D) surface morphology images were observed using atomic force microscopy (AFM, NanoMagnetics Instruments, Ankara, Turkey, ezAFM). Magnetic domain distribution was examined using magnetic force microscopy (MFM). AFM was operated in non-contact mode with three scanning repeats at RT to achieve average area evaluation. Surface roughness, represented by arithmetic mean deviation (Ra), was measured with a scanning size of 20 μm × 20 μm. The evaluation of low-frequency alternate-current magnetic susceptibility (χ_ac_) involved utilizing the MagQu χ_ac_ Quan II analyzer (MagQu, New Taipei City, Taiwan). Calibration included the application of an external magnetic field during the measurement of χ_ac_ using a standard sample. The analysis of χ_ac_ encompassed a frequency range of 10 Hz to 25,000 Hz, and χ_ac_ values were obtained from magnetization strength using a dedicated χ_ac_ analyzer. To maintain consistency and prevent demagnetization, all samples were kept at a uniform size of 0.5 cm × 0.5 cm, forming a square shape. Given that the exchange results are relative to the reference standard, χ_ac_ was expressed in arbitrary units (a.u.). Measurements employing χ_ac_ were crucial in establishing the correlation between magnetization and frequency, with the χ_ac_ analyzer determining the highest χ_ac_ at the optimal resonance frequency (f_res_). Contact angle measurements were executed using deionized (DI) water and glycerin. For precision, three replicates of each contact angle were taken, and their mean values were calculated. The determination of surface energy was performed using a contact angle measuring device (CAM-110 by Creating Nano Technologies in Tainan City, Taiwan) [32,33,34]. Moreover, the optical property was evaluated using a Spectro Smart Analyzer (OtO Photonics, Spectra Smart, Collimage, Taipei, Taiwan) with a visible light source spanning the wavelength range of 500–800 nm.

## 3. Results

### 3.1. X-ray Diffraction Pattern

**Figure 1:**
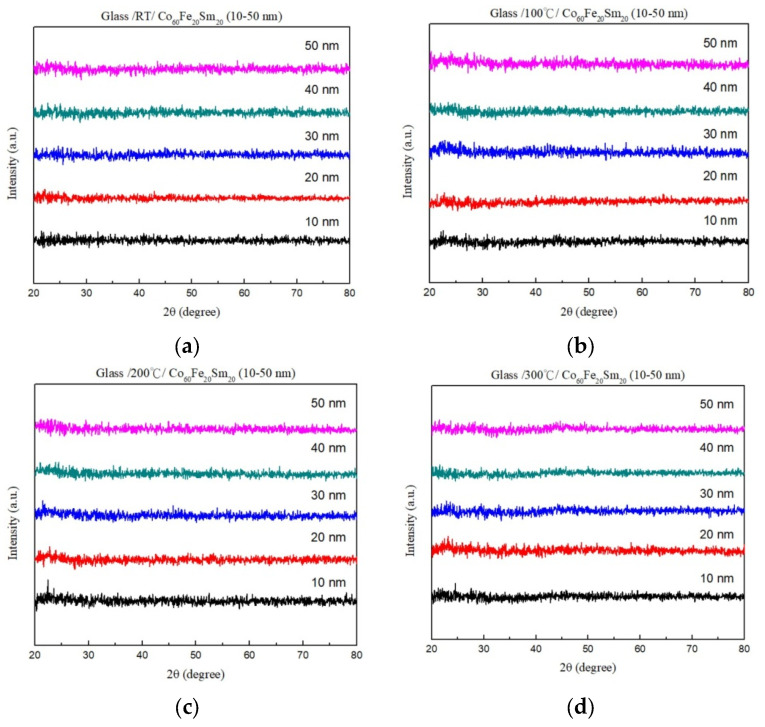
XRD patterns of Co_60_Fe_20_Sm_20_ films. (**a**) RT, (**b**) after annealing at 100 °C, (**c**) after annealing at 200 °C, (**d**) after annealing at 300 °C.

The XRD patterns in Figure 1a–d present the analysis of the Co_60_Fe_20_Sm_20_ thin films under the four conditions. Remarkably, these patterns did not display distinct characteristic peaks, highlighting the amorphous nature of the glass substrate. Several factors could be influencing this characteristic diffraction pattern. The absence of a crystalline structure in the films may be attributed to the insufficient thermal driving force required to facilitate the grain growth and glass substrate effect [35,36]. Furthermore, the study suggests that when the Sm content surpassed 13%, CoFeSm films transitioned into an amorphous state [37]. As a result, the sputter deposition of Co_60_Fe_20_Sm_20_ onto a glass substrate leads to a disorderly atomic structure.

**Figure 2:**
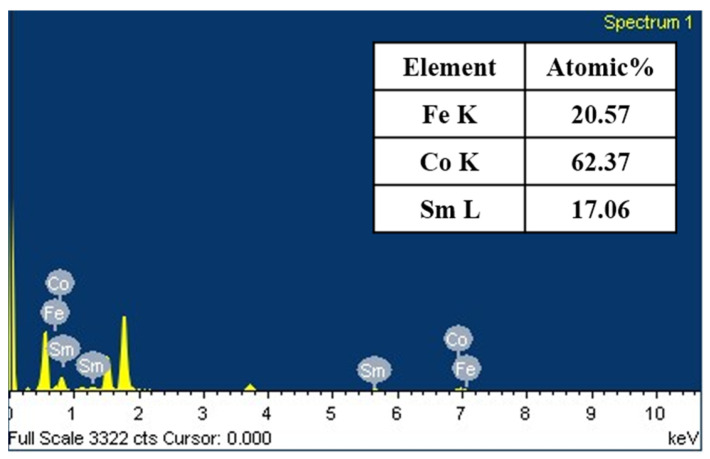
EDS element analysis of as-deposited Co_60_Fe_20_Sm_20_ thin films at 50 nm.

Figure 2 illustrates the energy dispersive X-ray spectroscopy (EDS) elemental analysis pattern for Co_60_Fe_20_Sm_20_ thin films with a thickness of 50 nm immediately after deposition. The EDS elemental analysis confirmed the presence of Co, Fe, and Sm. However, the results indicated that the Co, Fe, and Sm contents deviated slightly from the nominal stoichiometry of the sputtering alloy target, which was intended to be at 60 at.%, 20 at.%, and 20 at.%, respectively. This discrepancy between the target’s intended composition and its actual composition may be attributed to the influences of Ar ion bombardment and the ion angle during the sputter deposition process [38].

### 3.2. Electrical Property

**Figure 3:**
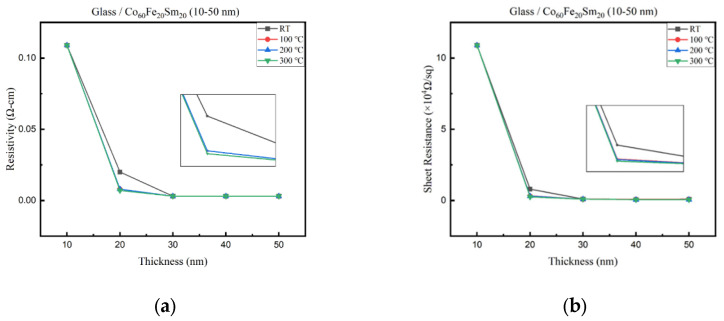
Electrical properties of CoFeSm films. (**a**) Resistivity and (**b**) sheet resistance.

Figure 3a,b investigate the variation in resistivity and sheet resistance of the thin films with changes in film thickness and annealing temperature. The results demonstrated a consistent reduction in resistivity and sheet resistance as film thickness and annealing temperature increase. Noteworthy was the Co_60_Fe_20_Sm_20_ annealed at 300 °C, with a 50 nm thickness, displaying the lowest resistivity and sheet resistance values at 0.003 (Ω-cm) and 0.06 (×10^4^ Ω/sq). The resistivity and sheet resistance of the thin films exhibited a clear decreasing trend with an increase in film thickness. This behavior is consistent with previous research findings in thin film materials [39]. As the film thickness increases, the number of conducting paths available for electron transport also increases. Consequently, the overall resistivity of the film decreases. The increased film thickness provides a greater volume of material for charge carriers to move through, effectively reducing the resistance encountered [40]. Annealing is known to improve the crystallinity and structural order of thin films. At higher temperatures, atoms in the film rearrange to form a more ordered and compact structure, thereby reducing defects and enhancing carrier mobility [41]. The annealing process can also assist in the removal of impurities and oxygen vacancies, which are common in as-deposited films. These impurities can act as scattering centers for charge carriers, leading to higher resistivity. Annealing helps in the healing of these defects, contributing to the observed reduction in resistivity and sheet resistance. Furthermore, annealing-induced stress relaxation within the film can enhance its electrical properties. Stress relief can promote better electron transport by reducing local strain-induced dislocations and defects, ultimately leading to lower resistivity and sheet resistance [42,43]. This study demonstrated a consistent reduction in resistivity and sheet resistance with increasing film thickness and annealing temperature. Annealing at higher temperatures enhances the film’s crystallinity, reduces defects, and relieves stress, collectively leading to improved electrical conductivity. Understanding these trends and underlying mechanisms is essential for optimizing thin film materials for various electronic applications.

### 3.3. Hardness and Young’s Modulus

**Figure 4:**
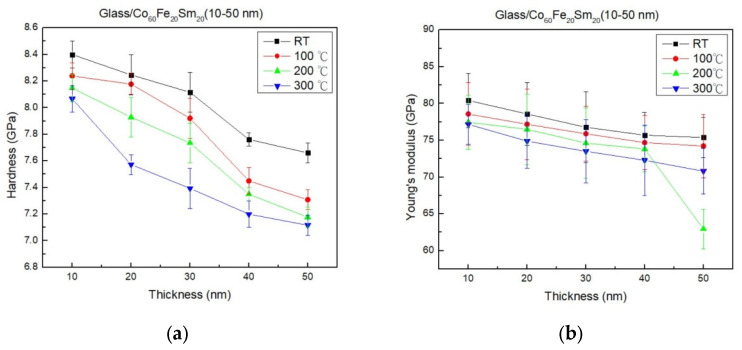
Hardness and Young’s modulus of CoFeSm films. (**a**) Hardness and (**b**) Young’s modulus.

Figure 4a,b reveals a clear trend where the hardness and Young’s modulus of CoFeSm thin films decreased with increasing film thickness and annealing temperature. The hardness and Young’s modulus of amorphous thin films exhibited a clear decreasing trend with an increase in film thickness. This trend is ascribed to size effects, an elevated defect density, or strain relaxation [44,45,46]. With an increase in the thickness of amorphous films, the material’s dimensions expand, giving rise to size effects. The dominance of surface and interfacial effects can cause materials to manifest altered mechanical properties, ultimately leading to lower hardness and Young’s modulus. Thicker amorphous films may exhibit a higher defect density or undergo strain relaxation, both of which can act as stress concentrators, resulting in a more compliant structure and, consequently, reduced mechanical properties. Annealing at elevated temperature results in a significant reduction in hardness and Young’s modulus. During annealing, the atoms in the film undergo rearrangement and structural transformation, leading to a more ordered and relaxed state [47]. This structural relaxation reduces internal stress and defects within the film, making it more compliant and less stiff. Additionally, annealing-induced diffusion and recrystallization can lead to the formation of a more crystalline and defect-free structure, further contributing to the reduction in hardness and Young’s modulus.

### 3.4. Magnetic Property

**Figure 5:**
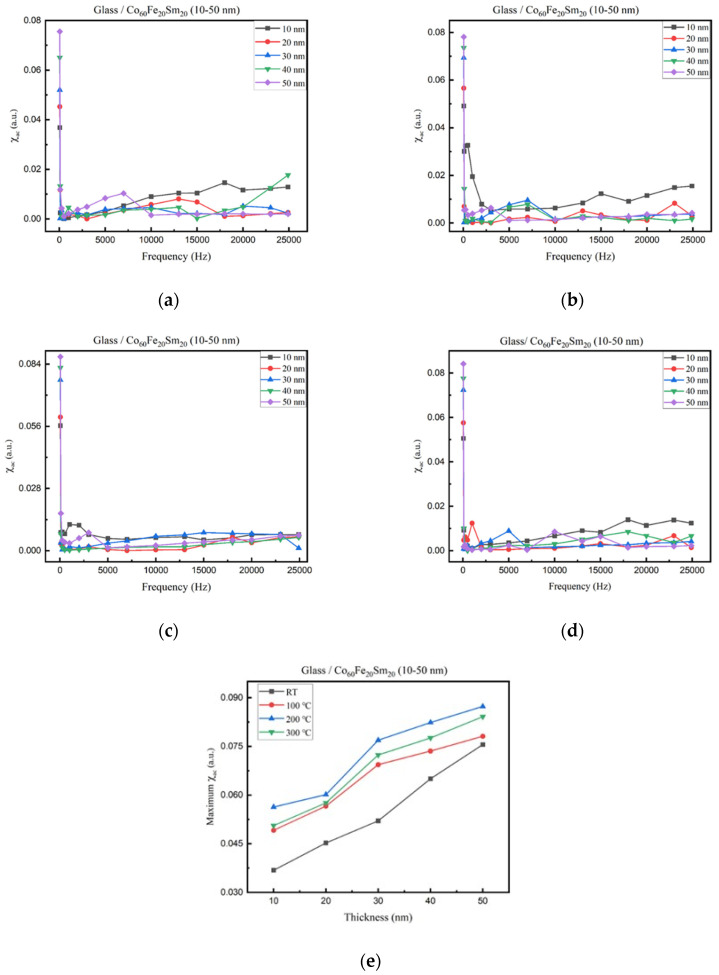
χ_ac_ of CoFeSm films. (**a**) RT, (**b**) after annealing at 100 °C, (**c**) after annealing at 200 °C, (**d**) after annealing at 300 °C, (**e**) maximum χ_ac_ value.

Figure 5a–d display a consistent trend in the low-frequency magnetic susceptibility (χ_ac_) values of CoFeSm thin films under different conditions. The graphs illustrate a progressive rise in χ_ac_ values for the Co_60_Fe_20_Sm_20_ films. Furthermore, as the frequency increased, there was a notable decrease in χ_ac_ values. Additionally, an interesting trend became apparent as the maximum χ_ac_ value increased with both film thickness and annealing temperature due to the thickness effect and smoother surface. This pattern is visually depicted in Figure 5e. A smoother surface with reduced roughness mitigated the impact on domain wall pinning, facilitating easier movement and thereby enhancing the χ_ac_ value [48,49]. Remarkably, the peak χ_ac_ value was attained at 200 °C, underscoring the substantial influence of annealing temperature on magnetic properties and its potential for modulation. This observation is supported by magnetic force microscopy (MFM) images, which converge on the optimal annealing temperature for CoFeSm thin films being 200 °C. In contrast, χ_ac_ values following annealing at 300 °C were diminished compared to those at 200 °C, likely due to an amplified thermal disturbance effect [50]. The maximum χ_ac_ value increased with growing thickness, attributed to the thickness effect [51].

### 3.5. Analysis of Contact Angle and Surface Energy

**Figure 6:**
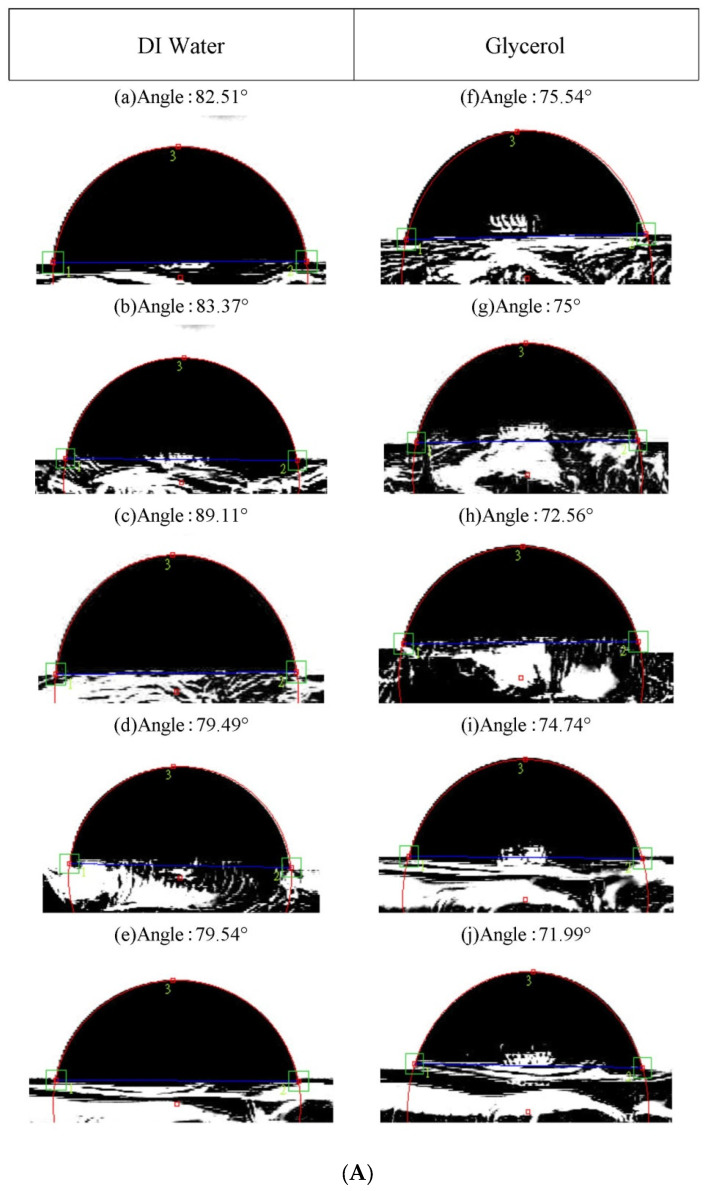

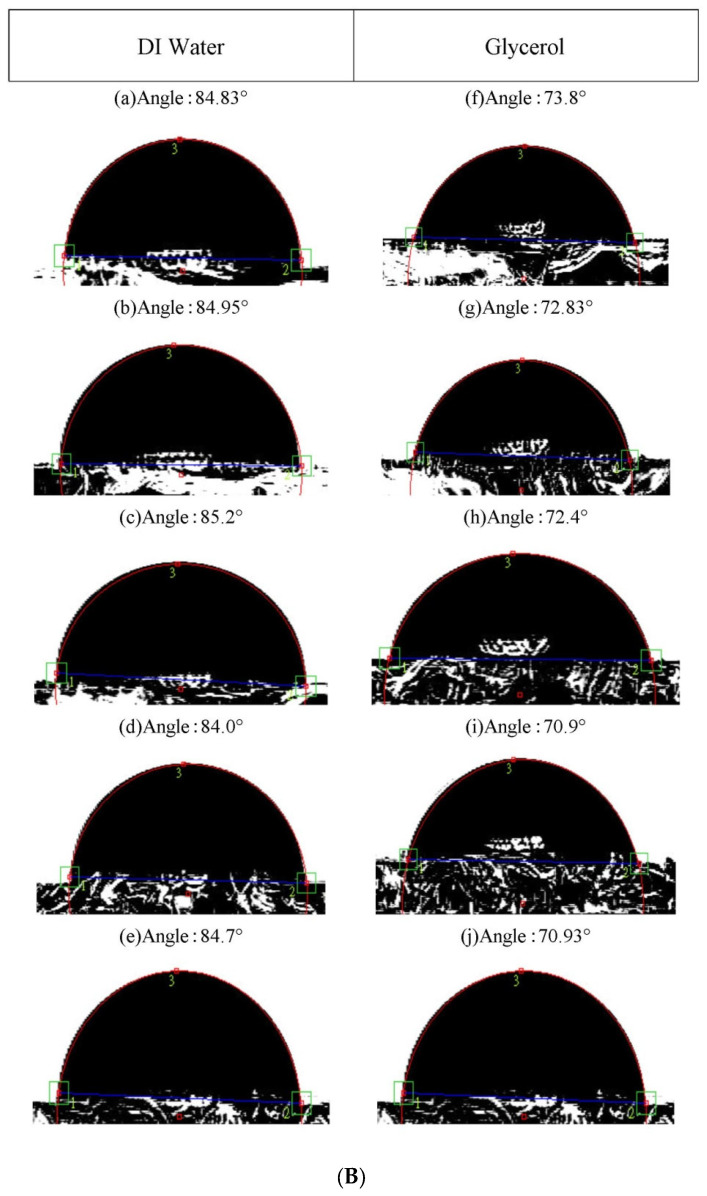

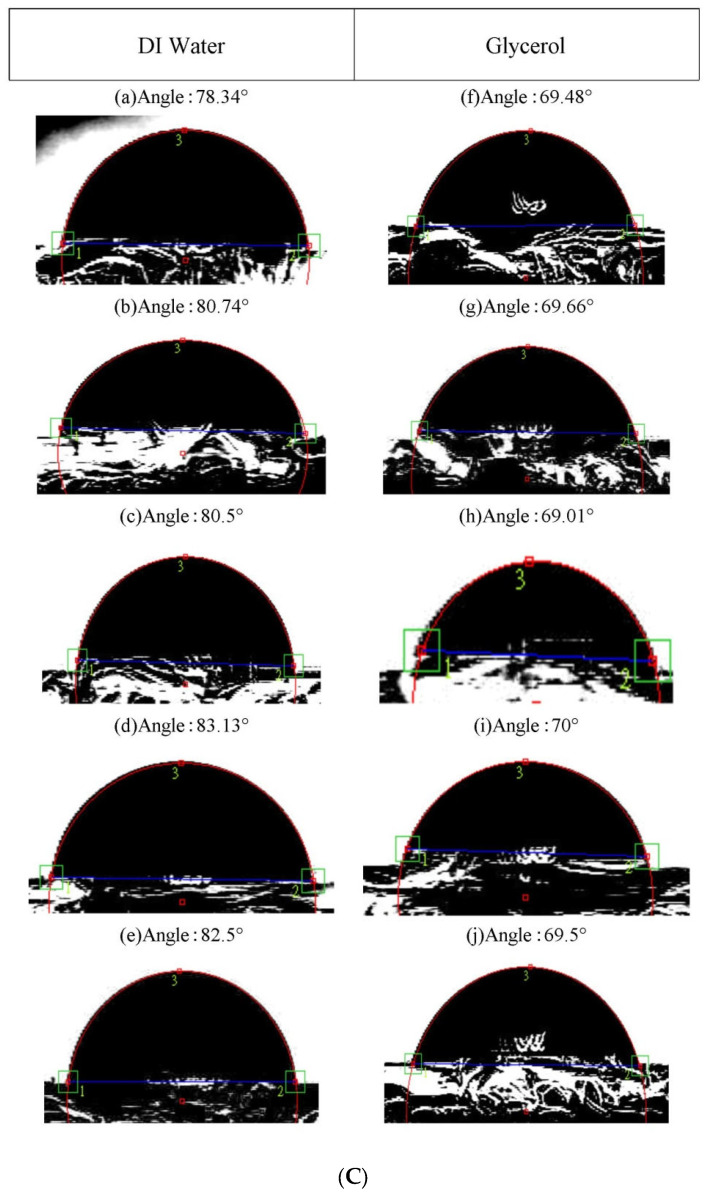

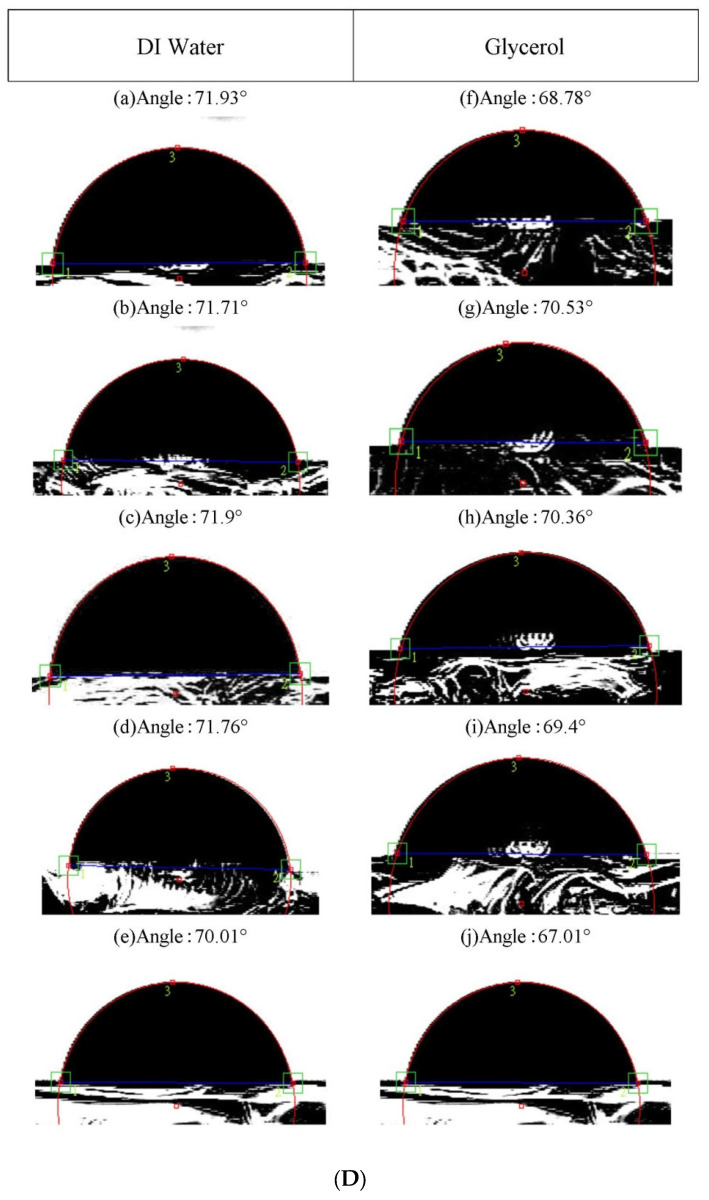
The images of contact angles under four various conditions: (**A**) RT, (**B**) after annealing at 100 °C, (**C**) after annealing at 200 °C, and (**D**) after annealing at 300 °C with DI water: (a) 10 nm, (b) 20 nm, (c) 30 nm, (d) 40 nm, and (e) 50 nm. Glycerol: (f) 10 nm, (g) 20 nm, (h) 30 nm, (i) 40 nm, and (j) 50 nm.

Figure 6A–D depict images illustrating contact angles (θ) under four distinct conditions. The contact angles of the films were measured using both DI water and glycerol. Notably, the Co_60_Fe_20_Sm_20_ films consistently exhibited contact angles of less than 90°, with the liquid drops appearing nearly spherical. These observations indicate that the films possess favorable hydrophilicity and wettability characteristics.

**Figure 7:**
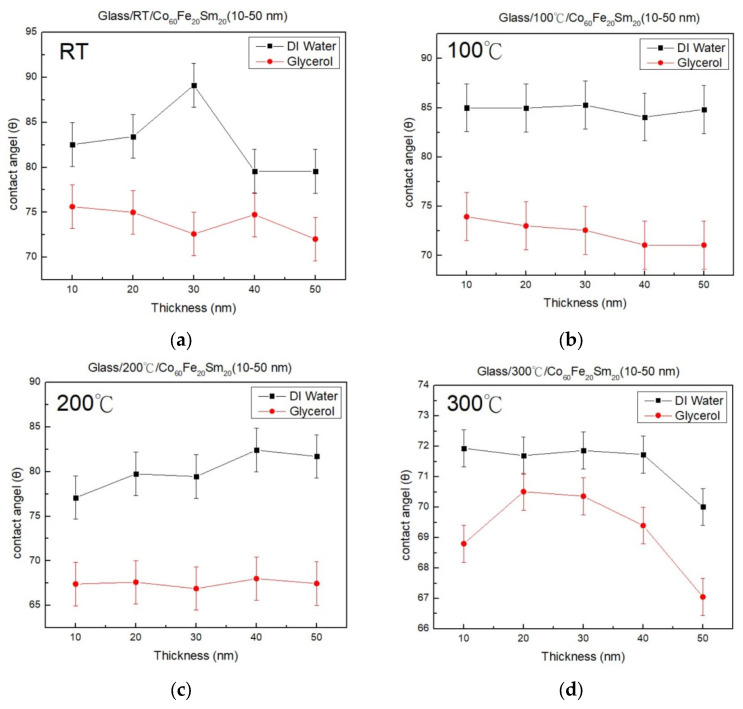
Contact angle of CoFeSm films. (**a**) RT, (**b**) after annealing at 100 °C, (**c**) after annealing at 200 °C, (**d**) after annealing at 300 °C.

Figure 7a–d present the results of contact angle measurements under four distinct conditions. This involved placing droplets of DI water and glycerol onto the surfaces of Co_60_Fe_20_Sm_20_ thin films and subsequently quantifying the resulting contact angles. The experimental data consistently indicate that the contact angles observed with DI water were of greater magnitude compared to those obtained with glycerol. In Figure 7a, an increasing trend in contact angle at room temperature (RT) is depicted within the thickness range of 10 nm to 30 nm, followed by a subsequent decrease within the range of 30 nm to 50 nm, specifically for the testing liquid DI water. Conversely, the results for glycerol exhibited the opposite trend. Figure 7b illustrates a declining trend in contact angle with both testing liquids as the thickness increased during annealing at 100 °C. Figure 7c presents an increasing contact angle trend with both testing liquids as the thickness increased during annealing at 200 °C. Figure 7d demonstrates a decreasing contact angle trend with DI water as the thickness increased during annealing at 300 °C. The contact angle exhibited an increasing trend from 10 to 20 nm, followed by a decrease from 20 to 50 nm, specifically for the testing liquid glycerol. Furthermore, the contact angles measured for both liquid types consistently fell within the range of 67 to 89°, remaining below 90°, indicative of the hydrophilic nature of the Co_60_Fe_20_Sm_20_ thin films. Additionally, the contact angles decreased with increasing thickness and annealing temperature. The reduction in contact angle with greater film thickness may be attributed to the larger surface area and reduced surface roughness, allowing for enhanced wetting and a subsequent decrease in contact angle. Thicker films offer a more continuous and smoother surface, thereby improving wetting characteristics [52,53]. Moreover, at elevated annealing temperatures, the film undergoes structural alterations that result in a smoother surface and improved wetting behavior, contributing to a decrease in contact angle. The heightened mobility of atoms and molecules at elevated temperatures further promotes better surface homogeneity [54,55]. In summary, this experiment underscores the favorable affinity of Co_60_Fe_20_Sm_20_ thin films for water-based liquids, showcasing their hydrophilic behavior.

**Figure 8:**
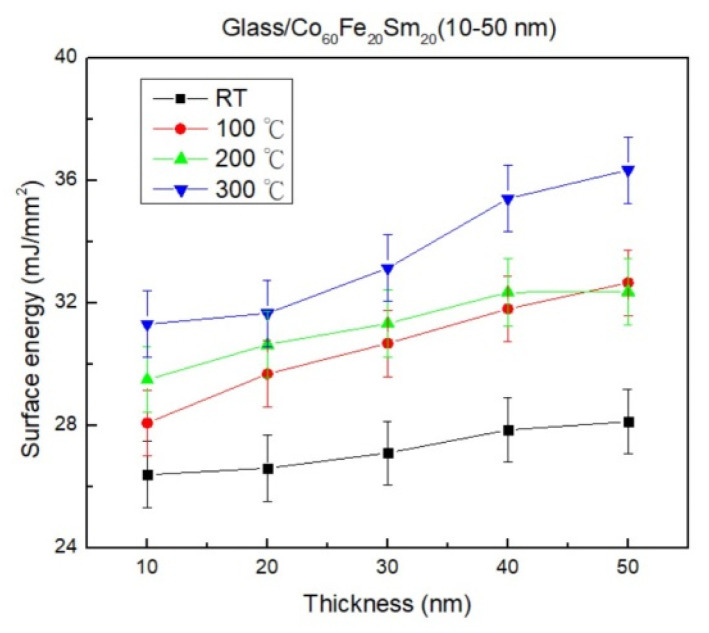
Surface energy of CoFeSm films.

The surface energy exhibited a proportional increase with both film thickness and annealing temperature, ranging from 26.3 to 36.4 mJ/mm^2^, as illustrated in Figure 8. This observation highlights that, particularly at an annealing temperature of 300 °C, Co_60_Fe_20_Sm_20_ thin film with a thickness of 50 nm manifests significantly heightened surface energy. The noticeable reduction in contact angle reflected a consistent rising trend in surface energy, signifying enhanced adhesion of the thin film. Importantly, a previous study established a robust linear correlation between water contact angle and material surface energy, wherein a smaller contact angle corresponded to higher surface energy. The escalation in surface energy with increasing film thickness is attributed to the expanded surface area of thicker films, enabling more interactions and greater energy at the surface [56]. Thicker films offer an increased number of active surface sites, consequently enhancing surface energy. Additionally, at elevated annealing temperatures, the film undergoes structural modifications leading to an augmented surface energy. The heightened mobility of atoms and molecules at higher temperatures contributes to a surface that is more energetically favorable [57].

### 3.6. Optical Experiment Results and Analysis

**Figure 9:**
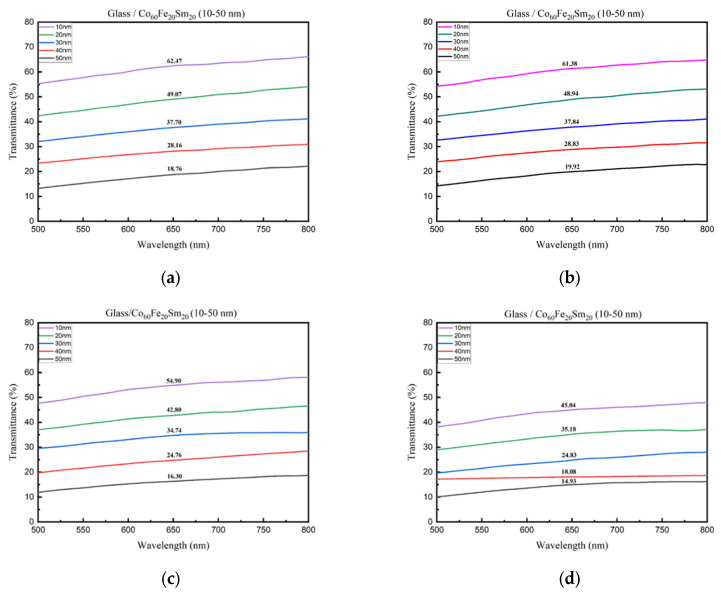
Transmittance (%) of CoFeSm thin films. (**a**) RT, (**b**) after annealing at 100 °C, (**c**) after annealing at 200 °C, (**d**) after annealing at 300 °C.

In Figure 9a–d, the transmittance (%) is depicted for varying film thicknesses under four distinct conditions, revealing a discernible pattern. The transmittance (%) consistently diminished with increasing film thickness and annealing temperature. Particularly in the visible light wavelength spectrum, the absorption intensity of Co_60_Fe_20_Sm_20_ thin films amplified progressively with film thickness. This trend highlights an inverse relationship between absorption and transmittance. The empirical data validate the notion that both thickness-dependent and interface-related factors hinder photon signal transmission through the film, consequently diminishing transmittance and influencing the optical properties of the thin film. Additionally, the results consistently also demonstrated that transmittance rose with an increase in surface roughness, underscoring the importance of surface morphology in optical characteristics. The heightened transmittance with greater surface roughness may be ascribed to the multiple scattering and diffraction effects induced by surface irregularities. Surface roughness generates microstructures that scatter light in diverse directions, enabling more light to traverse the film and augmenting transmittance [58].

### 3.7. Surface Magnetic Domains of the Thin Film

**Figure 10:**
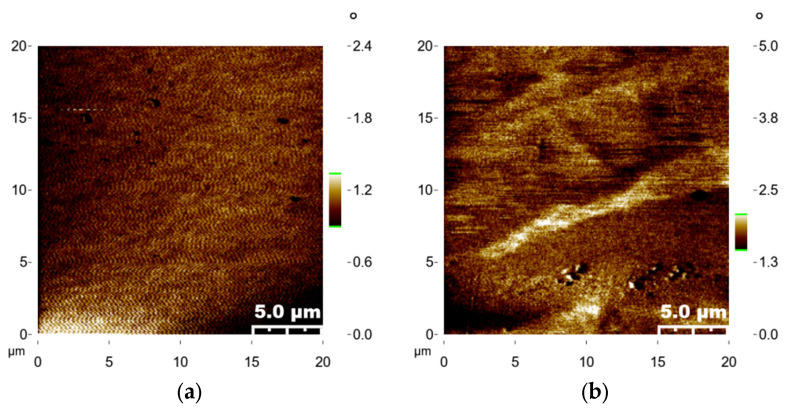

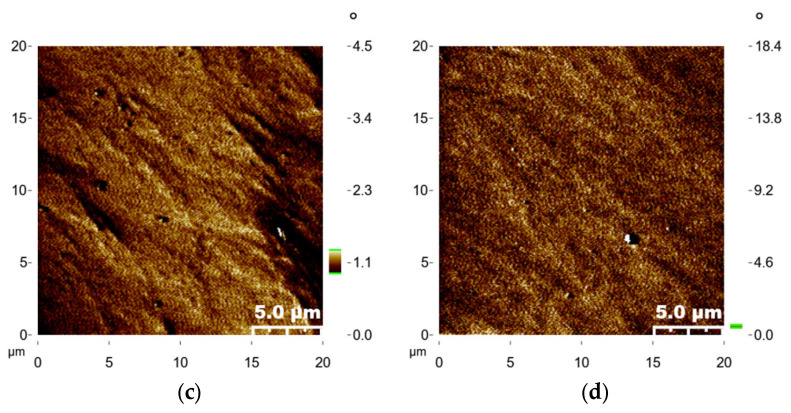
Surface magnetic domains of CoFeSm with a thickness of 50 nm. (**a**) RT, (**b**) after annealing at 100 °C, (**c**) after annealing at 200 °C, (**d**) after annealing at 300 °C.

In the magnetic domain images depicted in Figure 10a–d for the thin films, variations in brightness can be observed. Figure 10d stands out, showing a distinct decrease in brightness contrast within the magnetic domains, hinting at a reduction in the magnetic anisotropy. The scanning dimensions for the samples encompass an area of 20 µm × 20 µm. This decline is hypothesized to be linked to the presence of an antiferromagnetic layer on the film’s surface, aligning with the magnetic results and substantiating that the maximum χ_ac_ at 300 °C is smaller than that at 200 °C [59].

### 3.8. Surface Roughness and 3D Surface Topography Images of the Thin Film

**Figure 11:**
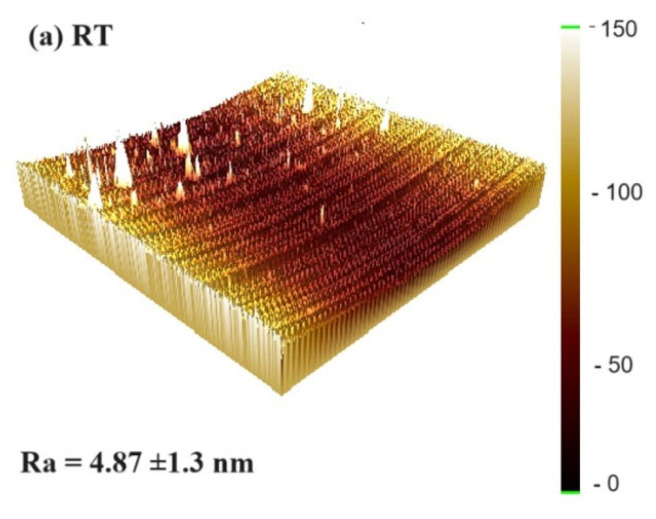

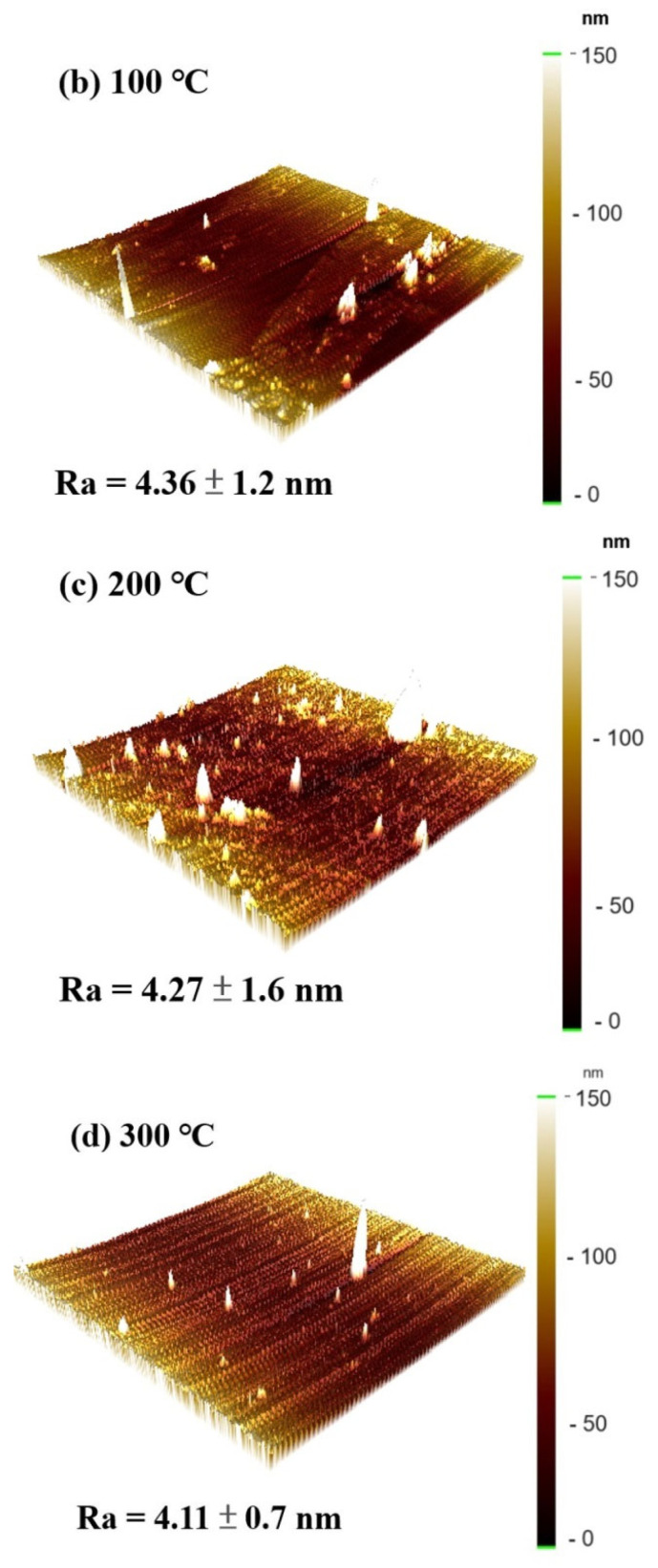
Surface roughness and 3D surface topography of the CoFeSm thin film with a thickness of 50 nm. (**a**) RT, (**b**) after annealing at 100 °C, (**c**) after annealing at 200 °C, (**d**) after annealing at 300 °C.

Figure 11a–d offer insights into the surface roughness and 3D surface topography of the thin film. An average roughness (Ra) of 50 nm was measured within a flat region, registering at 4.87 nm at RT and subsequently decreasing to 4.11 nm as the annealing temperature elevated to 300 °C. These findings imply that at lower sputtering power settings, surface protrusions may arise, causing variations and an increase in surface roughness. Annealing the thin film tends to result in a smoother surface [60]. The surface roughness of the CoFeSm films at different annealing temperatures significantly influences their magnetic, electrical, and adhesive properties. A smoother surface reduces the pinning effect on domain walls, facilitating their movement and enhancing the χ_ac_ value. Moreover, a smoother surface leads to a lower contact angle and higher surface energy. In contrast, rougher surfaces exhibit lower carrier conductivity, resulting in higher electrical resistance.

## 4. Conclusions

The research revealed that the properties of CoFeSm films, including their structure, electrical, magnetic, adhesive, and optical characteristics, were significantly influenced by both film thickness and annealing temperature. The absence of distinctive peaks in X-ray diffraction (XRD) analysis was attributed to insufficient growth-driving forces and the impact of the glass substrate. Electrical measurements demonstrated a decrease in resistivity and sheet resistance as film thickness increased and annealing temperatures rose. This reduction was due to hindered current-carrier transport in the amorphous structure. At higher annealing temperatures, the atoms in the film reorganized into a more ordered arrangement, resulting in improved carrier mobility, while impurity removal and defect reduction also played a role. These impurities could act as scattering centers for charge carriers, leading to increased resistivity. Nanoindentation analysis revealed that as film thickness increased, the hardness and Young’s modulus of the amorphous films decreased. This may be attributed to the influence of size effects, a higher defect density, and strain relaxation. Furthermore, the χ_ac_ values increased with both film thickness and annealing temperature, primarily due to the thickness effect and a smoother surface. A smoother surface reduced the domain wall-pinning effects, making it easier for domain walls to move and thus enhancing the χ_ac_ value. However, after annealing at 300 °C, a significant decrease in χ_ac_ values was observed, likely due to heightened thermal disturbance effects. Contact angles decreased with increasing thickness and annealing temperature, owing to a larger surface area and reduced surface roughness. Thicker films provided a larger, smoother surface that promoted better wetting. Higher annealing temperatures also contributed to a smoother surface, further reducing contact angles. The increased mobility of atoms and molecules at elevated temperatures further enhanced surface homogeneity. Both film thickness and interface-related factors hindered photon signal transmission through the film, reducing transmittance. Additionally, increasing surface roughness consistently led to higher transmittance, underscoring the significance of surface morphology in optical characteristics. This enhanced transmittance with greater surface roughness resulted from the scattering and diffraction effects caused by surface irregularities, creating microstructures that scattered light in various directions. The study highlighted the critical role of surface roughness in influencing magnetic, electrical, adhesive, and optical properties. Smoother surfaces were associated with reduced domain wall pinning, increased χac values, lower contact angles, higher surface energy, and enhanced carrier conductivity, thereby reducing electrical resistance. Optical transparency decreased with smoother surfaces. The optimal combination involved annealing at 300 °C with a film thickness of 50 nm, leading to superior adhesion, an enhanced χ_ac_ value, and reduced electrical resistance.

## Data Availability

Not applicable.
